# Diaqua-1κ*O*,2κ*O*-(2,2′-bi-1*H*-imidazole-1κ^2^ *N* ^3^,*N* ^3′^)(oxalato-2κ^2^ *O* ^1^,*O* ^2^)di-μ-oxido-κ^4^ *O*:*O*-dioxido-1κ*O*,2κ*O*-dimolyb­denum(V) trihydrate

**DOI:** 10.1107/S1600536809052003

**Published:** 2009-12-09

**Authors:** Xiutang Zhang, Peihai Wei, Congwen Shi, Bin Li, Bo Hu

**Affiliations:** a Advanced Material Institute of Research, Department of Chemistry and Chemical Engineering, ShanDong Institute of Education, Jinan, 250013, People’s Republic of China; b College of Chemistry and Chemical Engineering, Liaocheng University, Liaocheng, 252059, People’s Republic of China

## Abstract

In the title compound, [Mo_2_(C_2_O_4_)O_4_(C_6_H_6_N_4_)(H_2_O)_2_]·3H_2_O, the coordination polyhedra for both Mo(V) atoms consist of two bridging O atoms, two atoms of the chelating ligand (oxalate or diimidazole), a terminal O atom and one H_2_O mol­ecule. The two distorted octa­hedrally coordinated Mo(V) atoms are linked together *via* O—O edge-sharing and Mo—Mo inter­actions with a Mo—Mo bond length of 2.564 (5) Å. Uncoordinated water mol­ecules are situated in the voids of the crystal structure. N—H⋯O and O—H⋯O hydrogen bonding between the neutral mol­ecules and the water mol­ecules lead to a consolidation of the structure.

## Related literature

For background to polyoxometalates, see: Pope & Müller (1991 ▶). For polyoxometalates modified with amines, see: Zhang, Dou *et al.* (2009 ▶); Zhang, Wei *et al.* (2009 ▶).
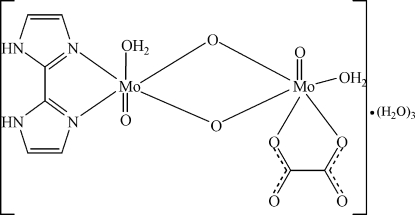


## Experimental

### 

#### Crystal data

[Mo_2_(C_2_O_4_)O_4_(C_6_H_6_N_4_)(H_2_O)_2_]·3H_2_O
*M* *_r_* = 568.13
Monoclinic, 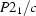

*a* = 10.7509 (16) Å
*b* = 14.517 (2) Å
*c* = 11.3661 (17) Å
β = 92.306 (2)°
*V* = 1772.4 (5) Å^3^
*Z* = 4
Mo *K*α radiation
μ = 1.49 mm^−1^
*T* = 273 K
0.12 × 0.10 × 0.08 mm

#### Data collection

Bruker APEXII CCD diffractometer
Absorption correction: multi-scan (*SADABS*; Bruker, 2001 ▶) *T*_min_ = 0.841, *T*_max_ = 0.890
11601 measured reflections
3099 independent reflections
2820 reflections with *I* > 2σ(*I*)
*R*_int_ = 0.020

#### Refinement

*R*[*F* ^2^ > 2σ(*F* ^2^)] = 0.021
*wR*(*F* ^2^) = 0.059
*S* = 1.00
3099 reflections
275 parameters
H atoms treated by a mixture of independent and constrained refinement
Δρ_max_ = 0.48 e Å^−3^
Δρ_min_ = −0.36 e Å^−3^

### 

Data collection: *APEX2* (Bruker, 2004 ▶); cell refinement: *SAINT-Plus* (Bruker, 2001 ▶); data reduction: *SAINT-Plus*; program(s) used to solve structure: *SHELXS97* (Sheldrick, 2008 ▶); program(s) used to refine structure: *SHELXL97* (Sheldrick, 2008 ▶); molecular graphics: *SHELXTL* (Sheldrick, 2008 ▶); software used to prepare material for publication: *SHELXTL*.

## Supplementary Material

Crystal structure: contains datablocks global, I. DOI: 10.1107/S1600536809052003/wm2286sup1.cif

Structure factors: contains datablocks I. DOI: 10.1107/S1600536809052003/wm2286Isup2.hkl

Additional supplementary materials:  crystallographic information; 3D view; checkCIF report

## Figures and Tables

**Table 1:** Selected bond lengths (Å)

Mo1—O8	1.68 (3)
Mo1—O7	1.94 (3)
Mo1—O6	1.94 (3)
Mo1—O1*W*	2.13 (3)
Mo1—N3	2.20 (4)
Mo1—N1	2.31 (4)
Mo2—O5	1.68 (3)
Mo2—O6	1.94 (3)
Mo2—O7	1.94 (3)
Mo2—O1	2.11 (3)
Mo2—O2*W*	2.16 (3)
Mo2—O4	2.23 (3)

**Table 2:** Hydrogen-bond geometry (Å, °)

*D*—H⋯*A*	*D*—H	H⋯*A*	*D*⋯*A*	*D*—H⋯*A*
N2—H2⋯O4^i^	0.86	2.02	2.84 (5)	160
N4—H4*A*⋯O3^i^	0.86	1.90	2.76 (5)	172
O1*W*—H1*W*⋯O6^ii^	0.8 (5)	1.9 (4)	2.66 (5)	168
O1*W*—H2*W*⋯O4*W*^iii^	0.8 (4)	1.8 (5)	2.56 (6)	170
O2*W*—H4*W*⋯O7^iv^	0.8 (4)	1.8 (5)	2.65 (5)	172
O3*W*—H5*W*⋯O2^v^	0.8 (4)	2.8 (7)	2.94 (7)	92
O5*W*—H9*W*⋯O3*W*^vi^	0.9 (5)	2.1 (6)	2.93 (7)	158
O2*W*—H3*W*⋯O3*W*	0.8 (2)	1.9 (3)	2.69 (7)	160

Symmetry codes: (i) 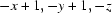; (ii) 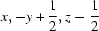; (iii) 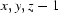; (iv) 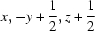; (v) 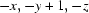; (vi) 
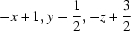
.

**Fig. 1.**
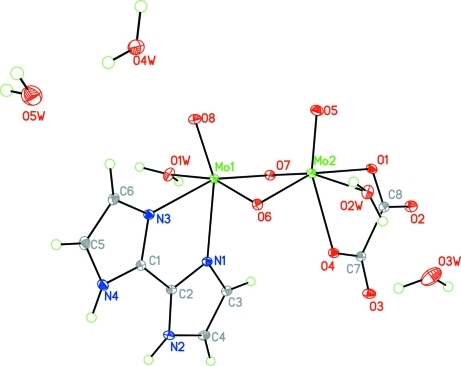
The asymmetric unit of the title compound with the atom-labelling scheme. Displacement ellipsoids are drawn at the 30% probability level; H atoms are given as spheres of arbitrary radius.

**Fig. 2.**
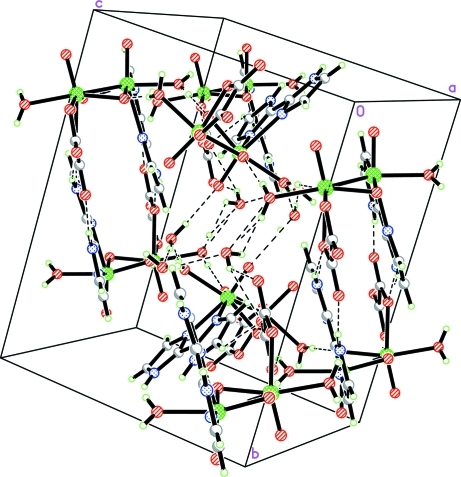
The packing diagram of the title compound with hydrogen bonds (dashed lines).

## supplementary crystallographic information

### Comment

The design and synthesis of polyoxometalates has attracted continuous research interest not only because of their appealing structural and topological novelties, but also due to their interesting optical, electronic, magnetic, and catalytic properties, as well as their potential medical applications (Pope *et al.*, 1991). In our group, organic amines, such as 3-(2-pyridyl)pyrazole and pyrazine, are used to effectively modify polyoxomolybdates (Zhang, Dou *et al.*, 2009); Zhang, Wei *et al.*, 2009). Here, we describe the synthesis and structural characterization of the title compound.

As shown in Figure 1, the asymmetric unit contains two Mo(V) ions, one of which is chelated by one diimidazole ligand, and the other chelated by one oxalate anion. Both Mo(V) ions are coordinated by one associated water molecule and one terminal oxygen atom. The two Mo(V) ions are linked together by two *µ*-oxygen atoms and by Mo—Mo bonding (2.564 (5) Å). Moreover, three uncoordinated water molecules are found in the voids of the crystal packing. Hydrogen bonding interactions between the Mo-containing molecule and water molecules further consolidates the structure (Fig. 2; Table 2).

### Experimental

A mixture of diimidazole (1 mmoL), molybdenum trioxide (1 mmoL), and oxalic acid (1 mmoL) in 10 ml distilled water were sealed in a 25 ml Teflon-lined stainless steel autoclave which was kept at 433 K for three days. Colorless crystals suitable for the X-ray diffraction study were obtained. Anal. Calc. for C_8_H_14_Mo_2_N_4_O_13_: C 16.96, H 2.47, N 9.89%; Found: C 16.85, H 2.40, N 9.78%.

### Refinement

All hydrogen atoms bound to C or N atoms were refined using a riding model with a distance C—H = 0.93 Å (N—H = 0.86 Å) and *U*_iso_ = 1.2*U*_eq_ (C, N). The H atoms of the water molecules were located from difference density maps and were refined with distance restraints of d(H–H) = 1.38 (2) Å, d(O—H) = 0.8 (2) Å, and with a fixed *U*_iso_ of 0.08 Å^2^.

### Crystal data

**Table:**

[Mo_2_(C_2_O_4_)O_4_(C_6_H_6_N_4_)(H_2_O)_2_]·3H_2_O	*F*(000) = 1120
*M**_r_* = 568.13	*D*_x_ = 2.129 Mg m^−^^3^
Monoclinic, *P*2_1_/*c*	Mo *K*α radiation, λ = 0.71073 Å
Hall symbol: -P 2ybc	Cell parameters from 3099 reflections
*a* = 10.7509 (16) Å	θ = 1.9–25.0°
*b* = 14.517 (2) Å	µ = 1.49 mm^−^^1^
*c* = 11.3661 (17) Å	*T* = 273 K
β = 92.306 (2)°	Block, colorless
*V* = 1772.4 (5) Å^3^	0.12 × 0.10 × 0.08 mm
*Z* = 4	

### Data collection

**Table:**

Bruker APEXII CCD diffractometer	3099 independent reflections
Radiation source: fine-focus sealed tube	2820 reflections with *I* > 2σ(*I*)
graphite	*R*_int_ = 0.020
phi and ω scans	θ_max_ = 25.0°, θ_min_ = 1.9°
Absorption correction: multi-scan (*SADABS*; Bruker, 2001)	*h* = −12→12
*T*_min_ = 0.841, *T*_max_ = 0.890	*k* = −17→17
11601 measured reflections	*l* = −13→13

### Refinement

**Table:**

Refinement on *F*^2^	Primary atom site location: structure-invariant direct methods
Least-squares matrix: full	Secondary atom site location: difference Fourier map
*R*[*F*^2^ > 2σ(*F*^2^)] = 0.021	Hydrogen site location: inferred from neighbouring sites
*wR*(*F*^2^) = 0.059	H atoms treated by a mixture of independent and constrained refinement
*S* = 1.00	*w* = 1/[σ^2^(*F*_o_^2^) + (0.036*P*)^2^ + 1.2494*P*] where *P* = (*F*_o_^2^ + 2*F*_c_^2^)/3
3099 reflections	(Δ/σ)_max_ = 0.001
275 parameters	Δρ_max_ = 0.48 e Å^−^^3^
0 restraints	Δρ_min_ = −0.36 e Å^−^^3^

### Special details

**Table:**

Geometry. All e.s.d.'s (except the e.s.d. in the dihedral angle between two l.s. planes) are estimated using the full covariance matrix. The cell e.s.d.'s are taken into account individually in the estimation of e.s.d.'s in distances, angles and torsion angles; correlations between e.s.d.'s in cell parameters are only used when they are defined by crystal symmetry. An approximate (isotropic) treatment of cell e.s.d.'s is used for estimating e.s.d.'s involving l.s. planes.
Refinement. Refinement of *F*^2^ against ALL reflections. The weighted *R*-factor *wR* and goodness of fit *S* are based on *F*^2^, conventional *R*-factors *R* are based on *F*, with *F* set to zero for negative *F*^2^. The threshold expression of *F*^2^ > σ(*F*^2^) is used only for calculating *R*-factors(gt) *etc*. and is not relevant to the choice of reflections for refinement. *R*-factors based on *F*^2^ are statistically about twice as large as those based on *F*, and *R*- factors based on ALL data will be even larger.

### Fractional atomic coordinates and isotropic or equivalent isotropic displacement parameters (Å^2^)

**Table:**

	*x*	*y*	*z*	*U*_iso_*/*U*_eq_	
Mo1	0.4186 (3)	0.2145 (3)	0.0209 (3)	0.0233 (14)	
Mo2	0.2035 (4)	0.2602 (3)	0.0911 (3)	0.0250 (14)	
C1	0.659 (4)	0.326 (3)	0.056 (4)	0.026 (10)	
C2	0.591 (4)	0.381 (3)	−0.030 (4)	0.025 (10)	
C3	0.436 (4)	0.411 (3)	−0.151 (4)	0.030 (10)	
H3	0.3598	0.4076	−0.1927	0.036*	
C4	0.523 (5)	0.478 (3)	−0.163 (4)	0.033 (11)	
H4	0.5168	0.5278	−0.2146	0.040*	
C5	0.788 (5)	0.267 (4)	0.188 (5)	0.038 (12)	
H5	0.8565	0.2586	0.2395	0.045*	
C6	0.691 (4)	0.208 (3)	0.170 (4)	0.033 (11)	
H6	0.6819	0.1520	0.2078	0.040*	
C7	0.108 (4)	0.427 (3)	−0.044 (4)	0.028 (10)	
C8	0.007 (4)	0.352 (3)	−0.060 (4)	0.030 (10)	
N1	0.479 (3)	0.351 (3)	−0.066 (3)	0.026 (8)	
N2	0.620 (4)	0.458 (3)	−0.087 (3)	0.029 (9)	
H2	0.6881	0.4889	−0.0777	0.035*	
N3	0.609 (4)	0.246 (3)	0.086 (3)	0.028 (9)	
N4	0.767 (3)	0.341 (3)	0.115 (3)	0.031 (9)	
H4A	0.8137	0.3886	0.1091	0.038*	
O1	0.028 (3)	0.279 (2)	0.004 (3)	0.032 (8)	
O2	−0.084 (3)	0.364 (3)	−0.125 (3)	0.042 (9)	
O3	0.100 (3)	0.497 (2)	−0.103 (3)	0.040 (8)	
O4	0.195 (3)	0.407 (2)	0.032 (3)	0.029 (7)	
O5	0.161 (3)	0.160 (2)	0.149 (3)	0.040 (8)	
O6	0.370 (3)	0.288 (2)	0.154 (3)	0.028 (7)	
O7	0.261 (3)	0.232 (2)	−0.065 (3)	0.028 (7)	
O8	0.431 (3)	0.106 (2)	0.070 (3)	0.037 (8)	
O1W	0.488 (3)	0.171 (3)	−0.143 (3)	0.038 (8)	
O2W	0.135 (3)	0.328 (2)	0.245 (3)	0.037 (8)	
O3W	0.078 (6)	0.499 (4)	0.315 (5)	0.081 (16)	
O4W	0.678 (4)	0.065 (3)	0.860 (4)	0.050 (10)	
O5W	0.905 (4)	0.104 (3)	0.965 (4)	0.065 (12)	
H1W	0.46 (5)	0.18 (5)	−0.21 (3)	0.080*	
H2W	0.55 (4)	0.14 (4)	−0.15 (5)	0.080*	
H3W	0.12 (6)	0.384 (14)	0.25 (3)	0.080*	
H4W	0.18 (6)	0.31 (4)	0.30 (4)	0.080*	
H7W	0.65 (5)	0.02 (3)	0.89 (6)	0.080*	
H8W	0.74 (4)	0.08 (4)	0.90 (5)	0.080*	
H9W	0.93 (6)	0.08 (5)	1.03 (3)	0.080*	
H10W	0.94 (6)	0.08 (5)	0.91 (3)	0.080*	
H5W	0.15 (2)	0.50 (6)	0.29 (6)	0.080*	
H6W	0.03 (5)	0.52 (6)	0.27 (5)	0.1 (4)*	

### Atomic displacement parameters (Å^2^)

**Table:**

	*U*^11^	*U*^22^	*U*^33^	*U*^12^	*U*^13^	*U*^23^
Mo1	0.028 (2)	0.022 (2)	0.020 (2)	−0.0018 (15)	−0.0015 (16)	0.0013 (15)
Mo2	0.026 (2)	0.027 (2)	0.022 (2)	−0.0032 (15)	−0.0012 (16)	0.0014 (15)
C1	0.03 (2)	0.03 (2)	0.02 (2)	0.000 (19)	0.001 (18)	−0.001 (19)
C2	0.03 (2)	0.02 (2)	0.02 (2)	−0.002 (18)	0.001 (18)	0.001 (18)
C3	0.03 (3)	0.03 (2)	0.03 (2)	0.00 (2)	−0.01 (2)	0.002 (19)
C4	0.04 (3)	0.03 (2)	0.03 (2)	0.00 (2)	0.00 (2)	0.01 (2)
C5	0.03 (3)	0.05 (3)	0.04 (3)	0.01 (2)	−0.01 (2)	0.01 (2)
C6	0.03 (3)	0.03 (3)	0.03 (3)	0.01 (2)	0.00 (2)	0.01 (2)
C7	0.03 (2)	0.03 (3)	0.02 (2)	0.00 (2)	0.002 (19)	−0.002 (19)
C8	0.03 (3)	0.03 (3)	0.03 (2)	0.00 (2)	0.00 (2)	0.00 (2)
N1	0.03 (2)	0.024 (19)	0.024 (19)	−0.001 (16)	−0.002 (16)	0.002 (15)
N2	0.03 (2)	0.03 (2)	0.03 (2)	−0.007 (17)	−0.001 (17)	0.003 (16)
N3	0.03 (2)	0.03 (2)	0.03 (2)	−0.001 (16)	−0.001 (17)	0.005 (16)
N4	0.03 (2)	0.03 (2)	0.03 (2)	−0.005 (17)	−0.003 (17)	0.005 (18)
O1	0.027 (17)	0.031 (18)	0.038 (19)	−0.006 (14)	−0.003 (15)	0.004 (15)
O2	0.038 (19)	0.04 (2)	0.05 (2)	−0.004 (16)	−0.019 (17)	0.000 (17)
O3	0.04 (2)	0.032 (19)	0.04 (2)	−0.007 (16)	−0.008 (16)	0.010 (16)
O4	0.027 (16)	0.027 (17)	0.031 (17)	−0.004 (13)	−0.005 (14)	0.000 (14)
O5	0.04 (2)	0.04 (2)	0.04 (2)	−0.007 (16)	0.004 (16)	0.009 (17)
O6	0.028 (17)	0.033 (18)	0.024 (16)	−0.002 (14)	−0.002 (13)	−0.002 (13)
O7	0.031 (17)	0.031 (17)	0.022 (16)	−0.003 (14)	−0.003 (13)	−0.001 (13)
O8	0.04 (2)	0.027 (18)	0.038 (19)	−0.001 (15)	−0.001 (16)	0.004 (15)
O1W	0.05 (2)	0.04 (2)	0.024 (17)	0.016 (17)	−0.002 (15)	−0.003 (16)
O2W	0.04 (2)	0.05 (2)	0.025 (17)	0.009 (17)	0.000 (15)	0.002 (15)
O3W	0.12 (4)	0.06 (3)	0.06 (3)	0.03 (3)	−0.02 (3)	0.00 (3)
O4W	0.04 (2)	0.04 (2)	0.07 (3)	−0.001 (18)	−0.01 (2)	0.01 (2)
O5W	0.06 (3)	0.06 (3)	0.07 (3)	−0.01 (2)	−0.01 (3)	0.00 (3)

### Geometric parameters (Å, °)

**Table:**

Mo1—O8	1.68 (3)	C5—C6	1.35 (7)
Mo1—O7	1.94 (3)	C5—N4	1.37 (6)
Mo1—O6	1.94 (3)	C5—H5	0.9300
Mo1—O1W	2.13 (3)	C6—N3	1.39 (6)
Mo1—N3	2.20 (4)	C6—H6	0.9300
Mo1—N1	2.31 (4)	C7—O3	1.22 (6)
Mo1—Mo2	2.564 (5)	C7—O4	1.28 (5)
Mo2—O5	1.68 (3)	C7—C8	1.55 (6)
Mo2—O6	1.94 (3)	C8—O2	1.22 (6)
Mo2—O7	1.94 (3)	C8—O1	1.29 (6)
Mo2—O1	2.11 (3)	N2—H2	0.8600
Mo2—O2W	2.16 (3)	N4—H4A	0.8600
Mo2—O4	2.23 (3)	O1W—H1W	0.8 (5)
C1—N3	1.33 (6)	O1W—H2W	0.8 (4)
C1—N4	1.34 (6)	O2W—H3W	0.82 (16)
C1—C2	1.44 (6)	O2W—H4W	0.8 (6)
C2—N1	1.33 (5)	O3W—H5W	0.8 (4)
C2—N2	1.33 (6)	O3W—H6W	0.8 (7)
C3—C4	1.35 (7)	O4W—H7W	0.8 (5)
C3—N1	1.37 (6)	O4W—H8W	0.8 (6)
C3—H3	0.9300	O5W—H9W	0.9 (5)
C4—N2	1.37 (6)	O5W—H10W	0.8 (5)
C4—H4	0.9300		
			
O8—Mo1—O7	110.2 (15)	N4—C1—C2	131 (4)
O8—Mo1—O6	106.3 (15)	N1—C2—N2	111 (4)
O7—Mo1—O6	93.4 (13)	N1—C2—C1	117 (4)
O8—Mo1—O1W	89.1 (16)	N2—C2—C1	132 (4)
O7—Mo1—O1W	85.9 (13)	C4—C3—N1	109 (4)
O6—Mo1—O1W	163.7 (14)	C4—C3—H3	125.7
O8—Mo1—N3	91.4 (15)	N1—C3—H3	125.7
O7—Mo1—N3	158.0 (14)	C3—C4—N2	107 (4)
O6—Mo1—N3	84.3 (14)	C3—C4—H4	126.3
O1W—Mo1—N3	90.2 (14)	N2—C4—H4	126.3
O8—Mo1—N1	157.9 (15)	C6—C5—N4	107 (4)
O7—Mo1—N1	85.8 (13)	C6—C5—H5	126.4
O6—Mo1—N1	87.1 (13)	N4—C5—H5	126.4
O1W—Mo1—N1	76.6 (14)	C5—C6—N3	109 (4)
N3—Mo1—N1	72.2 (13)	C5—C6—H6	125.7
O8—Mo1—Mo2	101.5 (12)	N3—C6—H6	125.7
O7—Mo1—Mo2	48.7 (10)	O3—C7—O4	127 (4)
O6—Mo1—Mo2	48.6 (9)	O3—C7—C8	119 (4)
O1W—Mo1—Mo2	134.5 (10)	O4—C7—C8	114 (4)
N3—Mo1—Mo2	132.9 (10)	O2—C8—O1	125 (4)
N1—Mo1—Mo2	100.5 (9)	O2—C8—C7	121 (4)
O5—Mo2—O6	107.5 (15)	O1—C8—C7	114 (4)
O5—Mo2—O7	106.2 (16)	C2—N1—C3	106 (4)
O6—Mo2—O7	93.4 (13)	C2—N1—Mo1	115 (3)
O5—Mo2—O1	92.7 (15)	C3—N1—Mo1	139 (3)
O6—Mo2—O1	159.4 (13)	C2—N2—C4	107 (4)
O7—Mo2—O1	84.9 (13)	C2—N2—H2	126.5
O5—Mo2—O2W	88.3 (16)	C4—N2—H2	126.5
O6—Mo2—O2W	86.8 (13)	C1—N3—C6	106 (4)
O7—Mo2—O2W	164.7 (13)	C1—N3—Mo1	118 (3)
O1—Mo2—O2W	89.6 (13)	C6—N3—Mo1	135 (3)
O5—Mo2—O4	160.2 (15)	C1—N4—C5	107 (4)
O6—Mo2—O4	86.4 (12)	C1—N4—H4A	126.3
O7—Mo2—O4	86.5 (12)	C5—N4—H4A	126.3
O1—Mo2—O4	73.0 (11)	C8—O1—Mo2	120 (3)
O2W—Mo2—O4	78.3 (12)	C7—O4—Mo2	116 (3)
O5—Mo2—Mo1	99.3 (12)	Mo2—O6—Mo1	82.7 (12)
O6—Mo2—Mo1	48.7 (9)	Mo1—O7—Mo2	82.7 (12)
O7—Mo2—Mo1	48.6 (9)	H1W—O1W—H2W	106.00
O1—Mo2—Mo1	133.5 (10)	H3W—O2W—H4W	112.00
O2W—Mo2—Mo1	135.2 (9)	H5W—O3W—H6W	112.00
O4—Mo2—Mo1	100.4 (8)	H7W—O4W—H8W	107.00
N3—C1—N4	111 (4)	H9W—O5W—H10W	111.00
N3—C1—C2	118 (4)		

### Hydrogen-bond geometry (Å, °)

**Table:**

*D*—H···*A*	*D*—H	H···*A*	*D*···*A*	*D*—H···*A*
N2—H2···O4^i^	0.86	2.02	2.84 (5)	160
N4—H4A···O3^i^	0.86	1.90	2.76 (5)	172
O1W—H1W···O6^ii^	0.8 (5)	1.9 (4)	2.66 (5)	168
O1W—H2W···O4W^iii^	0.8 (4)	1.8 (5)	2.56 (6)	170
O2W—H4W···O7^iv^	0.8 (4)	1.8 (5)	2.65 (5)	172
O3W—H5W···O2^v^	0.8 (4)	2.8 (7)	2.94 (7)	92
O5W—H9W···O3W^vi^	0.9 (5)	2.1 (6)	2.93 (7)	158
O2W—H3W···O3W	0.8 (2)	1.9 (3)	2.69 (7)	160

Symmetry codes: (i) −*x*+1, −*y*+1, −*z*; (ii) *x*, −*y*+1/2, *z*−1/2; (iii) *x*, *y*, *z*−1; (iv) *x*, −*y*+1/2, *z*+1/2; (v) −*x*, −*y*+1, −*z*; (vi) −*x*+1, *y*−1/2, −*z*+3/2.
